# Supplementary material for: A retrospective analysis thyroid function and ultrasonography in a group of subjects with lepramatous leprosy in Turkey
Source: PLoS Negl Trop Dis. 2024 Jul 18;18(7):e0012344. doi: 10.1371/journal.pntd.0012344 (PMC11288423; doi:10.1371/journal.pntd.0012344)
Supplement: Attachment — Submitted filename: PLOS revision letter-z.docx [file pntd.0012344.s001.docx]

Dear Editor,

We are thankful for your and reviewers valuable evaluating for our article. We corrected and rearranged this article as reviewers requests. Every requests was performed and marked as red characters in the text.

We would be pleased if this article was found suitable for publication in your journal.

Kind Regards

Corresponding Author

Zeynep Ozkan, Assoc. Prof.

Usak University Medical Faculty Department Of Surgery

1-The population didn't clearly  described, how to confirm the study population  were previously diagnosed with lepramatous leprosy ( what type of diagnosed?).

Population was described clearly. (These patients were diagnosed by these criteria; detection of acid-fastbacilli in slit skin smears, and the histopathological examination of skin biopsies and typical dermal appearances caused by thickened nerves.

The patients who were included in the study were previously diagnosed in the LeprosyWard of our hospital and were followed for a long time. This ward is a continuation of the leprosy hospital, which was established during the leprosy fight that started approximately 80 years ago in our country, and the patients in this ward are those with chronic follow-up. The patients are not currently receiving active treatment and are only receiving social care services because of the sequelae of the disease)

2- A sample size of 17 is relatively small. While it's possible to conduct analyses, including t-tests, with this sample size, the results may have limited generalizability to the population. Small samplesizes can lead to less precise estimates and may not adequately. With a small sample size, violations of these assumptions can have a larger impact on the validity of the results. With small sample sizes, there's an increased risk of both Type I (false positive) and Type II (false negative) errors. Researcher sshould be cautious when interpreting results and consider the potential for error due to the small sample size.

Correct statistical analys is used to support conclusions but statistical analysis assumption is not explained well.

Although we do not have any data at hand, the prevalence of leprosy is very low in our region and no new patients have been registered to the Leprosy Center in our hospital in the last 8years. Therefore, our sample size was limited to 17 patients. These patients consisted of advanced age patients due to the very low incidence of new leprosy. We were arrangement our conclusi on this condition.

Thyroid diseases occur 5 times more frequently in women than in men in the general population. For this reason, the data of the patients included in the study group(Thyroid-stimulating hormone (TSH), freetriiodothyronine (FT3), freethyroxine (FT4) anti-thyroidperoxidase (Anti-Tpo), antithyroglobulin (Anti-Tg) andthyroglobulin (Tg) thyroidantibodies)were analyzed comparatively in terms of male and female gender.

3-Ethical or regulatory requirements being met:

Ethics CommitteeApproval: The ethics committee approval for the study (dated 29.04.2020 and numbered 2020/07-25) was obtained from the Non-Invasive Research Ethics Committee of Firat University, Elazig. All procedures performed in the study involving human participants were in accordance with the ethical standards of the institutional and/or national research committee and with the 1964 Helsinki declaration and its later amendments or comparable ethical standards.

4-In Materials and methods section it says "All patients had received any medication for leprosy and were follow up only.

These patients were diagnosed by these criteria; detection of acid-fastbacilli in slit skin smears, and the histopathological examination of skin biopsies and typicald ermal appearances caused by thickened nerves.

The patients who were included in the study were previously diagnosed in the LeprosyWard of our hospital and were followedf or a long time. This ward is a continuation of the leprosy hospital, which was established during th eleprosy fight that started approximately 80 years ago in our country, and the patients in this ward are those with chronic follow-up. The patients are not currently receiving active treatment and are only receiving social care services because of the sequelae of the disease.

5- The results did not clearly and completely presented with  the important variables  ( not include all socio-demography , family history....and others factors or variables:

Since the detailed characteristics of the patients, such as sociodemographic structures, family history, etc. were not related to the primary target of our study (the purpose of the study was to investigate thyroid functions and gland morphology and tissue characteristics of the thyroid gland in leprosy patients using ultrasonography (US) and laboratory techniques), they were not investigated and not discussed in the results section.

6- The limitations of analysisc learly  not described all issues

Limitation was written in separate session.

7- Include graphs in addition to tables

A pie chart graphic was added.

8-Requesting major revision bya ddressing  increase sample size by using more than 5 years data if available, include important variables for description and analysis.

Although we do not have any data at hand, the prevalence of leprosy is very low in our region and no new patients have been registered to the Leprosy Center in our hospital in the last 8 years. Therefore, our sample size was limited to 17 patients. These patients consisted of advanced age patients due to the very low incidence of new leprosy. We rearranged our conclusion this condition.
